# Prevalence of polycystic ovary syndrome in patients with type 2 diabetes: A systematic review and meta-analysis

**DOI:** 10.3389/fendo.2022.980405

**Published:** 2022-08-31

**Authors:** Caiyi Long, Haoyue Feng, Wen Duan, Xin Chen, Yuemeng Zhao, Ying Lan, Rensong Yue

**Affiliations:** ^1^ Hospital of Chengdu University of Traditional Chinese Medicine, Chengdu, China; ^2^ Chengdu University of Traditional Chinese Medicine, Chengdu, China

**Keywords:** Type 2 diabetes mellitus, polycystic ovary syndrome, prevalence, systematic review, meta-analysis

## Abstract

**Purpose:**

With type 2 diabetes mellitus (T2DM) occurring at a younger age, a greater number of women with T2DM experience reproductive health problems. The prevalence of polycystic ovary syndrome (PCOS), a common reproductive disease associated with T2DM, remains unknown in women with T2DM. This systematic review and meta-analysis aimed to determine the prevalence of PCOS in women with T2DM.

**Methods:**

Stata 15.1 was used to perform a meta-analysis on the prevalence of PCOS in patients with T2DM included in this study. Additionally, a narrative review of the effects of different diagnostic methods, obesity, state, and other factors on the prevalence of PCOS was conducted.

**Results:**

Meta-analysis showed that the overall prevalence of PCOS in women with T2DM was approximately 21%. Subgroup analysis showed that the incidence of PCOS in female patients aged 25-45 years was higher than that in female patients aged < 25 years. The prevalence of PCOS in obese women was 14%, which was lower than that in normal weight women and normal weight or overweight or obese women. Women with T2DM in Oceania had the highest incidence of PCOS, followed by those in Europe and Asia; women with T2DM in North America had the lowest incidence. In terms of PCOS diagnostic standards, the prevalence of PCOS diagnosed by the National Institutes of Health was the lowest. The prevalence of PCOS diagnosed on the basis of clinical symptoms and biochemical characteristics was the highest, and the prevalence of PCOS diagnosed on the basis of medical records was 20%.

**Conclusions:**

PCOS is a common disease in female patients with T2DM. The prevalence of PCOS in women with T2DM at childbearing age was higher than that in adolescent females. Women with T2DM at childbearing age should pay attention to the screening and prevention of PCOS to avoid the hazards of PCOS to reproductive health.

**Systematic review registration:**

PROSPERO, identifier CRD42022318657.

## Introduction

Diabetes mellitus (DM) is a major public health concern worldwide. According to statistics (1), approximately 537 million adults (20-79 years old) worldwide will develop DM in 2021, and the disease tends to occur at a younger age. At present, more patients are developing type 2 DM (T2DM) at a younger age, and female patients with T2DM are affected by the disease for a longer time during the reproductive period. Reproductive dysfunction in female patients with T2DM is gradually aggravating (2–4). Cytopathological changes in the uterine epithelium and basement membrane are progressively worsened by the hyperglycemic environment, which results in the progressive atrophy of cellular adipose tissue and promotes reproductive degeneration (5). It has been reported that irregular menstruation occurs in nearly 1/5 of women with T2DM (6). The fertility odds ratio has decreased by approximately 36% (7), and perinatal mortality and birth defect rates have increased (8). Attention should be paid to the reproductive health of female patients with T2DM since it affects pregnancy and the health of the offspring.

Polycystic ovary syndrome (PCOS) is a common endocrine and metabolic reproductive disease associated with various metabolic, cardiovascular, and psychological factors (9, 10) that affects approximately 7% of women of childbearing age worldwide (11). PCOS is characterized by oligoovulation or anovulation, hyperandrogenemia, irregular menstruation, and polycystic ovaries diagnosed by ultrasound (12). As a common endocrine and metabolic disease, T2DM is closely associated with PCOS. Compared with patients without T2DM, patients with T2DM are more likely to show clinical Tchanges, such as menstrual disorders and polycystic ovaries (13) (14),. This may be related to the increase in cellular androgen levels induced by the cooperation of insulin and luteinizing hormone. Furthermore, endogenous insulin resistance in type 2 diabetes and hyperinsulinemia can stimulate ovarian granulosa cells, which together promote the growth and number of small follicles (15, 16), subsequently resulting in PCOS. PCOS and T2DM share certain similarities in clinical, biochemical, and metabolic characteristics. There is also a high degree of overlap between patients with PCOS or T2DM (17–20). T2DM complicated by PCOS may result in critical injuries to the reproductive system. Except for a decrease in fertility and infertility (21–23), T2DM patients with PCOS have a higher risk of preterm birth (24). Insulin resistance exists in both diseases, which may induce endometrial dysfunction and placental abnormalities, subsequently resulting in miscarriage (25, 26). Previous studies have found that adult women with PCOS have an 8- to 18-fold increased risk of T2DM (18). However, evidence supporting the prevalence of PCOS in women with T2DM is lacking. Whether PCOS is a highly prevalent and non-routinely identified complication in women with T2DM requires further investigation.

The aims of this systematic review and meta-analysis were as follows (1): to clarify the prevalence of PCOS in female patients with T2DM (2), to assess the effects of multiple variables, including age, diagnostic criteria for PCOS, body mass index (BMI), state, T2DM duration, and T2DM treatment on the prevalence, and (3) to analyze the risk of PCOS in patients with T2DM at childbearing age, provide individualized screening strategies for timely diagnosis and treatment, delay the occurrence and development of the disease, and promote self-screening and management among this population.

## Materials and methods

### Systematic review protocol and registration

This study was registered in PROSPERO (ID: CRD42022318657). Institutional Review Board approval and informed consent were not obtained because the data were anonymous and publicly available. This study was drafted and reported based on the guidelines of a meta-analysis of observational studies in epidemiology (MOOSE) (27, 28).

### Literature search

The PubMed, Embase, Web of Science, and Cochrane Library databases were searched for studies. The main terms used in the search were “Polycystic ovary syndrome”, “Ovary polycystic disease”, “diabetes mellitus, type 2”, and “Non insulin dependent diabetes mellitus”. The specific search strategies are presented in **Tables 1** **–** **4** of **Supplementary Material 1** (29). References in previous systematic reviews and meta-analyses with similar topics were reviewed during the initial screening for supplementary information. During the screening of the full text of the studies, the references of the eligible studies were also reviewed for supplementary information. No published studies were retrieved. The retrieval period was from the establishment of the database until March 20, 2022. The language used in this study was English.

### Inclusion and exclusion criteria

To satisfy the requirements of this analysis and reduce selection bias, eligible studies met the following inclusion criteria: 1. The study was a cross-sectional or cohort study. 2. Study participants were female patients diagnosed with T2DM according to the reference diagnostic criteria. 3. Primary outcome measures included quantitative data from patients with PCOS. 4. The study did not include clinical interventions. If there was an intervention in the study, data before the intervention were selected. The exclusion criteria were as follows: 1. Duplication or literature with similar data (the literature with the most detailed data should be included) 2. The study participants were special populations, including pregnant women and patients with other diseases that result in excess androgen (e.g., Cushing's syndrome, hyperprolactinemia, thyroid dysfunction, and tumors) 3. Studies with incomplete data. 4. Studies that only reported polycystic ovary-like changes, polycystic ovaries, or hyperandrogenemia, without a definitive diagnosis of PCOS.

### Study selection, data extraction, and quality assessment

The included studies were independently screened and crosschecked by two researchers (CYL and YL) who majored in related specialties. If there was any dissent, a third specialist (RSY) assessed the results and provided solutions. The titles and abstracts of the studies were reviewed for initial screening, and subsequently, the full text was reviewed to determine whether the study could be included. Two researchers used unified criteria for data extraction, including the first author, publication year, country, study design, age, BMI, PCOS diagnostic method, total sample size, quantitative data on PCOS prevalence in participants, duration of T2DM, and treatments for T2DM.

The influence of each study on the results was determined using a sensitivity analysis. The assessment tool, which included 11 items specific for studies on prevalence, was used to assess the risk of bias (30). Among the items, the last was a summary item, with scores of > 8 as low risk, 6-8 as moderate risk, and ≤ 5 as high risk.

The US Agency for Healthcare Research and Quality (AHRQ; http://www.ncbi.nlm.nih.gov/books/NBK35156/) assessment checklist, which included 11 items, was used to evaluate the quality of the cross-sectional studies (31). “Yes”, “no”, or “not reported” were used to answer these items. Only “yes” was scored 1; “no” and “not reported” were scored 0. A score of 0-3 was considered as low quality, 4-6 as medium quality, and 8-11 was considered as high quality.

### Statistical analysis and outcomes

Stata 15.1 software was used for statistical analysis. A fixed- or random-effects model was selected based on the level of heterogeneity in the analysis. The results of the *I^2^* test were used to quantify heterogeneity. When the heterogeneity was large (*P* < 0.1, *I^2^* > 50%), a random-effects model was used; when the heterogeneity was small (*P* ≥ 0.1, *I^2^* ≤ 50%), a fixed-effects model was used. A 95% confidence interval (CI) was used as the effect scale. *P* < 0.05 was considered statistically significant. For *I^2^* > 75%, heterogeneity was considered high (32). Subgroup analysis or sensitivity analysis was used to determine the source of significantly high heterogeneity.

The primary outcome measure in this study was PCOS prevalence. Subgroup analyses were performed according to age, geographic region, BMI, and PCOS diagnostic criteria to investigate the role of multiple factors on the prevalence and the source of heterogeneity. Age was divided into two subgroups according to fertility (< 25 and 25-45 years old). Geographical areas were grouped according to the countries in which the states were located. BMI was grouped according to World Health Organization diagnostic criteria; individuals with BMI ranged from 18.5 to 24.9 kg/m^2^ were normal, ≥ 25 kg/m^2^ were overweight, and ≥ 30 kg/m^2^ were obese. Since BMI might vary over a large range in certain studies, the mean ± standard deviation of BMI of 18.5-25.0 kg/m^2^ was defined as normal, and ≥ 30 kg/m^2^ was defined as abnormal. If the BMI values belonged to two or more groups (normal, obese, or overweight), it was categorized as a mixed group.

## Results

### Study selection

**Figure 1:**
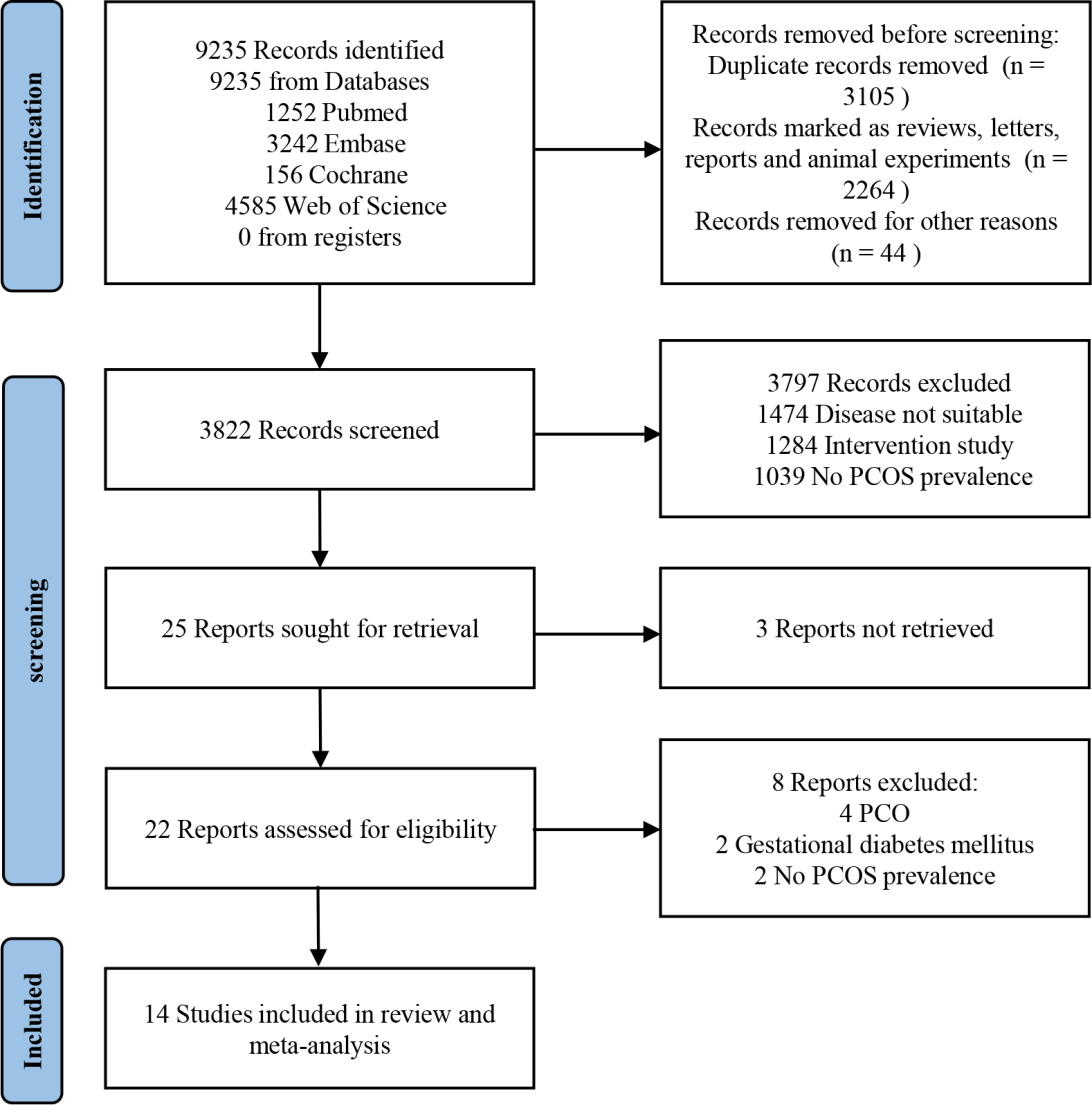
Study Flow Diagram.

In total, 9235 articles were retrieved. After excluding duplicates, animal experiments, conferences, reports, and letters, 3822 articles were selected. Based on titles and abstracts, 3797 articles were screened. After screening the full text, 14 trials were identified (14, 33–45) (**Figure 1**).

### Study characteristics

Finally, 14 trials, published between 2001 and 2016, were selected. A total of 1389 participants, aged between 9.9 and 75 years, covering Asia (14, 34–36, 39, 45), North America (33, 38, 42, 44), Europe (40, 41, 43), and Oceania (four states) (37), were studied. Most of the included studies were retrospective, and no randomized clinical trials were documented. Among these studies, four did not report BMI (38, 42, 44, 45), whereas others reported the BMI values between 18.01 and 44.1 kg/m^2^. Eight studies reported the duration of T2DM (33–35, 37, 39, 40, 43, 44). The longest duration of T2DM was 14.9 years, and its prevalence was the highest (37%) (37). The shortest duration of T2DM was 0 years, and its prevalence was among the lowest (8.5%) (44). Eight trials reported data on the treatment methods of T2DM (14, 36, 38–42, 45), including self-management, such as diet management and exercise, oral administration of drugs, and insulin treatment. However, the relationship between different treatment methods and PCOS prevalence was not reported. The basic characteristics are summarized in **Table 1**.

**Table 1:** Characteristics of included studies.

Study	Continent	Country	Design	Age	BMI	Duration of diabetes(years)	Treatment of T2DM	Method of PCOS assessment	Total	Event	Risk of bias	AHRQ
Sim2016	Oceania	Australia	RC	62(23-75)	34(9.9)	14.9	NR	Medical records	171	64	6	8
Mirzaei2008	Asia	Iran	PC	37.7 ± 5.22	26.48 ± 4.10	NR	Glibenclamide, metformin, or on diet alone	Clinical features	92	18	6	5
Amini2008	Asia	Iran	PC	39.29 ± 4.42	30.23 ± 5.01	5.92 ± 4.18	NR	NIH	157	13	7	6
Kelestimur2006	Asia	Turkey	RC	38.9 ± 0.5	31.8 ± 0.6	NR	Diet-controlled or an oral antidiabetic agent or insulin treatment	NIH	92	4	4	7
Zargar2005	Asia	India	PC	36.19 ± 4.37	25.42 ± 3.2	3.69 ± 1.95	NR	NIH	105	39	5	5
Peppard2001	North America	USA	RC	35.27 ± 2.32	40.3 ± 3.8	6.96 ± 1.66	NR	NIH	30	8	8	8
Amed2011	North America	Canada	RC	13.7(13.5-13.8)a	NR	NR	Insulin, lifestyle counseling, and insulin plus lifestyle counseling	Medical records	130	19	6	5
Shield2009	Europe	UK	PC	13.6(9.9-16.8)	32.5(18.7-56.2)	1	Diet alone, insulin alone, metformin ± other oral drugs, insulin and metformin, sulphonylurea ± other oral drugs	Clinical features	101	34	9	7
Balasanthiran2011	Europe	UK	RC	<25 years a	21.2(3.19)a	5.4(3.09)a	NR	Clinical features	27	6	2	1
Wilmot2010	Europe	UK	RC	<35 years a	33.7 ± 7.6a	NR	Metformin, insulin, sulfonylurea, and diet alone	NR	120	34	3	1
Amed2012	North America	Canada	RC	13.69(13.3-14.27)	NR	0	NR	Medical records	130	11	6	5
Amutha2012	Asia	India	RC	22.2 ± 9.7a	24.9 ± 5.0	5.94(0.48)a	OHAs, insulin and OHAs, diet and exercise	Clinical features	195	45	6	2
Zdravkovic2004	North America	Canada	RC	13.5(2.2)a	NR	NR	OHAs, insulin, diet, and exercise	Clinical features	26	6	9	3
Ramachandran2003	Asia	India	PC	17.23 ± 5.28	NR	NR	Metformin, glyclazide, glibenclamide, glipizide or insulin	Medical records	13	1	3	3

RC, retrospective cohort; PC, prospective cohort; NR, not reported; NIH, National Institutes of Health; a, value is representative of the entire cohort of the study including male patients; OHAs, oral hypoglycemic agent; BMI, body mass index; PCOS, polycystic ovary syndrome; T2DM, type 2 diabetes mellitus; AHRQ, Agency for Healthcare Research and Quality.

### Level of evidence

The AHRQ scale for assessing cross-sectional research quality was used to evaluate quality. As presented in **Supplementary Material 2**, five studies were rated as low-quality (38, 39, 41, 43, 45), seven studies were of moderate quality (14, 34–36, 40, 42, 44), and two were of high quality (33, 37). None of the studies reported the time interval for patient identification. Only one article included follow-up information (40) and only two explained the reasons for missing data (14, 33).

### Prevalence of PCOS in T2DM

**Figure 2:**
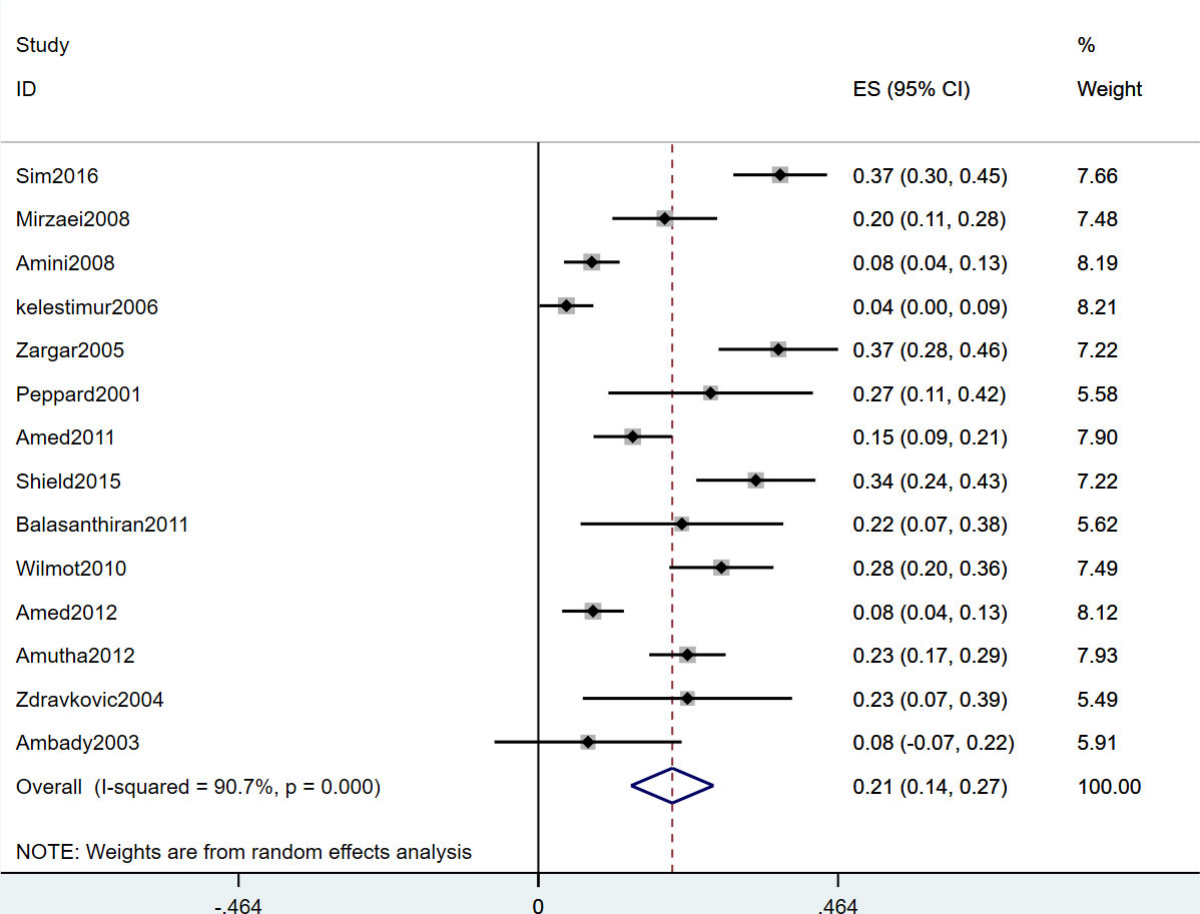
Forest Plot Showing Prevalence of PCOS in Patients With T2DM.

Owing to the high heterogeneity (*I^2^* = 90.7%, *P* = 0.000), a random-effects model was used for the analysis. The analysis showed that the overall prevalence of PCOS in patients with T2DM was 21% (95% CI: 0.14-0.27) (**Figure 2**).

### Subgroup analyses

The effects of age, BMI, state, and diagnostic criteria for PCOS on prevalence were explored through subgroup analyses (**Table 2**).

**Table 2:** Prevalence of including studies as per study and design characteristics.

Category	Subgroup	No. of studies	Prevalence% (95% CI)	*I^2^ *	*P*
Total		14	0.21 (0.14-0.27)	90.7	< 0.001
Age (years)					< 0.001
	<25	6	0.18 (0.09-0.26)	80.5	
	25-45	6	0.20 (0.10-0.30)	92.5	
BMI					< 0.001
	Normal	1	0.22 (0.07-0.38)		
	Abnormal	2	0.14 (-0.08-0.36)	86	
	Mixed	7	0.27 (0.17-0.36)	92.1	
	NR	4	0.12 (0.07-0.17)	37	
State					< 0.001
	Asia	6	0.17 (0.07-0.26)	91.8	
	Europe	3	0.30 (0.24-0.35)	0	
	North America	4	0.15 (0.08-0.22)	62	
	Oceania	1	0.37 (0.30-0.45)		
Diagnostic criteria for PCOS					< 0.001
	NIH	4	0.18 (0.05-0.30)	93.3	
	Clinical features	5	0.24 (0.19-0.29)	27.6	
	Medical records	5	0.20 (0.08-0.31)	92.3	

PCOS, polycystic ovary syndrome; BMI, body mass index; NR, not reported; NIH, National Institutes of Health; 95% CI, 95% confidence interval.

The prevalence in female patients aged 25-45 years and < 25 years was 20% (95% CI: 0.10-0.30) and 18% (95% CI: 0.09-0.26) (*P* < 0.001), respectively. The prevalence of PCOS in women with T2DM at childbearing age was significantly higher in adolescents. The prevalence in Oceania, Europe, Asia, and North America was 37% (95% CI: 0.30-0.45), 30% (95% CI: 0.24-0.35), 17% (95% CI: 0.07-0.26), and 15% (95% CI: 0.08-0.22), respectively (*P* < 0.01). The prevalence was the highest in Oceania. In terms of body weight, the prevalence in obese women, normal weight women, and normal or overweight or obese women was 14% (95% CI: -0.08-0.36), 22% (95% CI: 0.07-0.38), and 27% (95% CI: 0.17-0.36), respectively (*P* < 0.001). The prevalence of PCOS was the lowest among obese women. When stratified by the diagnostic criteria for PCOS, the prevalence of clinical symptoms and biochemical characteristics was the highest at 24% (95% CI: 0.19-0.29). National Institutes of Health (NIH) and medical records showed a prevalence of 18% (95% CI: 0.05-0.30) and 20% (95% CI: 0.08-0.31), respectively (*P* < 0.001).

Although we summarized the duration and treatment of T2DM mentioned in the included studies during data collection, only eight items were quantitative data, and the format was not uniform. Thus, a subgroup analysis was not performed for these factors.

### Sensitivity analyses

**Figure 3:**
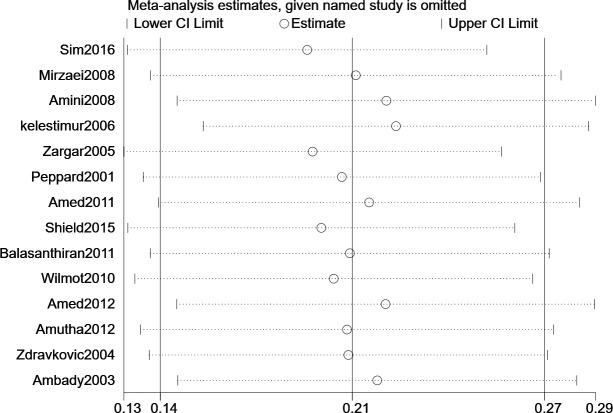
Sensitivity Analysis.

Sensitivity analyses were performed to investigate the effects of individual studies on the results. All results were robust (**Figure 3**).

### Risk of bias

As shown in **Supplementary Material 3**, the risk of bias assessment indicated that five studies were high-risk (14, 34, 41, 43, 45), seven studies were moderate-risk (33, 35–37, 39, 42, 44), and two studies were low-risk (38, 40). The mean risk score for these studies was 5.71 ± 2.16. Through bias analysis, it was found that in only three studies, the target population was representative of the national population (40, 42, 44), and more than half of the studies did not report the sample selection method for population selection. Most studies conducted direct data collection and unified collection patterns were observed.

### Publication bias

**Figure 4:**
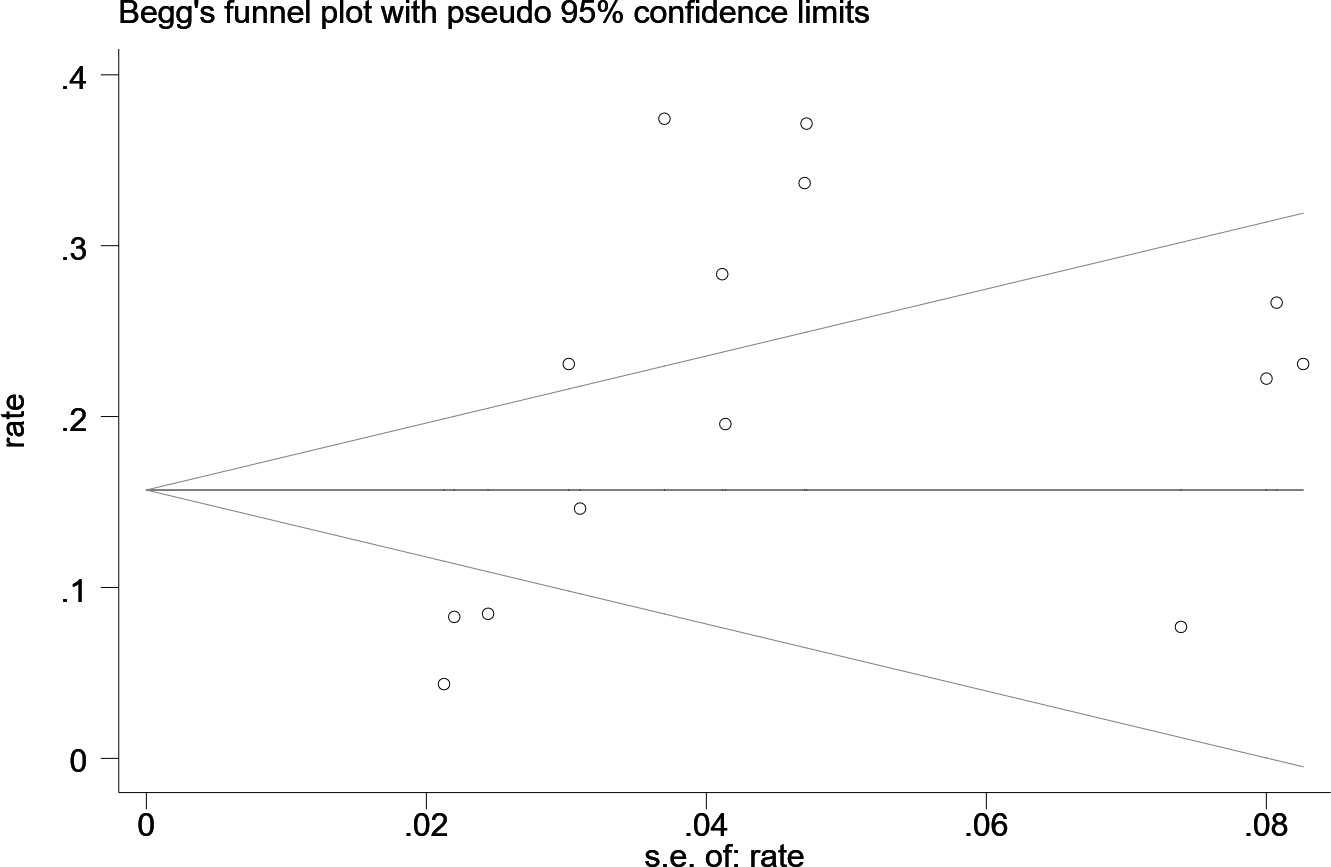
Begg’s publication bias plot.

Statistical assessment (Begg’s test, *P* = 0.155) suggested no publication bias in the studies (**Figure 4**).

## Discussion

Women with T2DM generally have a higher BMI (46), insulin resistance at an earlier age, and hyperinsulinemia (47) than women without T2DM, which may contribute to the increased prevalence of PCOS. The incidence of PCOS has increased owing to the occurrence of T2DM at a younger age, which may become a serious public health challenge. Our study analyzed PCOS prevalence in all age groups, especially in female T2DM patients of childbearing age, which comprised a larger population. This study is the first meta-analysis to investigate the prevalence of PCOS in women with T2DM of all ages worldwide and to suggest that nearly 1/5 of female patients with T2DM might develop PCOS, which is 2-4 times higher than that in women without T2DM (48) and similar to the prevalence of PCOS in women with type 1 DM (49). PCOS in female patients with T2DM is a highly prevalent complication that has not been routinely identified and screened. This should be noted by the public.

Our study investigated the potentially distorting factors, including age, state, BMI, and different diagnostic methods for PCOS, to provide evidence for the effective prevention of the disease.

The first pregnancy in women generally occurs at around 25 years of age (50), and women over 45 years of age have a higher risk of pregnancy. Therefore, the age of the participants in this study was limited to 25-45 years to determine the prevalence of PCOS. Because the ovarian function is closely related to age (51, 52), there is a sudden reduction in ovarian volume and a significant decrease in oocyte quality after 35 years of age (53), and the prevalence of PCOS increases with age (54). A higher prevalence of PCOS increases the risk of spontaneous abortion (21). Thus, combined with the results of our study, we recommend that women with T2DM at childbearing age should undergo PCOS screening as soon as possible to prevent the harm of the disease to reproduction.

The prevalence of PCOS varies significantly under different diagnostic criteria (55, 56), and uncertainty in diagnosis is accompanied by potential psychological harm due to misdiagnosis or overdiagnosis (57). The internationally recognized diagnostic criteria for PCOS include the 1990 NIH (58), the 2004 European Society for Human Reproduction and Embryology/American Society for Reproductive Medicine (59), and the 2006 Androgen Excess and PCOS Society (AE-PCOS) standards (60). Among these, ovarian polycystic changes are not mentioned in the NIH standard, while AE-PCOS mainly emphasizes that the clinical or biochemical characteristics of hyperandrogenemia must be considered. This study found that the prevalence was highest when the disease was diagnosed based on clinical symptoms, biochemistry, and ovarian ultrasound results and lowest when the disease was diagnosed based on NIH standard. These results were consistent with those of previous studies (48), which may be related to the inclusion of ovarian morphology in the diagnosis in former studies (55). This study also reflected the effects of different diagnostic criteria on the prevalence of PCOS. Therefore, there is an urgent need to resolve the uncertainty in diagnostic criteria for clinical diagnosis.

In this study, the order of prevalence in different continents was similar to the results of a previous study (48), and the specific values increased. The prevalence in Oceania was nearly doubled, which may be associated with the high prevalence of T2DM (61). Another reason may be that the hypertrichosis score (Ferriman–Gallwey) was included in the diagnostic criteria. Although T2DM is also highly prevalent in Asia (62), the prevalence of PCOS is lower than in other states due to a lower incidence of hypertrichosis and low BMI values (63). The prevalence of hypertrichosis in the Oceanian population was 21.2%, which was much higher than that in women in other regions (64, 65); thus, the prevalence of PCOS was also higher. These results were similar to the general trends reported in the present study. At the same time, few related studies have been conducted in Oceania, and only one study was included. Further studies are required to confirm these findings. Previous studies have also found that PCOS is closely related to family inheritance (66), and the influence of different races on PCOS has been confirmed (67, 68). Geographic isolation and differences in environmental conditions also affect PCOS prevalence in different states (67). Therefore, it is necessary to develop targeted screening measures for patients with T2DM in different states to prevent misdiagnosis and overdiagnosis.

Obesity is a common risk factor for both T2DM and PCOS. Some studies have shown that obesity in patients with T2DM could aggravate insulin resistance and hyperinsulinemia, leading to the onset of PCOS. However, some studies have also found that the onset of PCOS may not be related to BMI (69–71). A cross-sectional study found that there was a significant difference in insulin resistance between the PCOS and control groups, but there was no significant difference in the visceral, abdominal, and hip-waist fat ratio (72). Among the included studies, two trials classified into the obese subgroup were from Asia and North America (14, 33), and both used NIH as the diagnostic criteria, which might account for the lower prevalence. Although this study showed a lower prevalence of obesity, it may have been influenced by geographical differences, diagnostic criteria, and other factors. Further research is required to elucidate the relationship between obesity and the prevalence of PCOS in women with T2DM.

In this systematic review and meta-analysis, the following limitations in the included studies were found. First, the publication year of the included studies was early, and the latest literature was published in 2016, which may not reflect the current prevalence. Second, there was substantial heterogeneity among studies. On the one hand, because of the diversity in PCOS diagnostic criteria and regions, heterogeneity cannot be ruled out by subgroup analysis. On another hand, due to the lack of original data and inconsistency of data, this study failed to analyze the potential relationship between the course and treatment mode of T2DM and the prevalence of PCOS. Third, due to the limited number of articles and the differences in ethnicity, eating habits, climate in different countries, and the impact of the epidemic and the economy, the BMI diagnosis of obesity is different. This study was first formulated according to international unified standards; however, the bias of the results caused by the unified diagnosis cannot be excluded, and this study will continue to track the possible consequences of this difference. Fourth, among the included studies, only one case was conducted in Oceania, and there was a lack of relevant studies on the prevalence of PCOS in women with T2DM in South America. It is not possible to comprehensively analyze the differences in the prevalence of PCOS among women with T2DM in different states worldwide. Further multi-center and large-scale studies are required to evaluate the prevalence of PCOS in women with T2DM.

## Conclusion

This systematic review and meta-analysis found that about 1/5 of women with T2DM suffered from PCOS, which was much higher than that of women without T2DM. The prevalence of PCOS in women with T2DM at childbearing age was higher than that in adolescent women. Therefore, women with T2DM at childbearing age need to pay more attention to the screening and prevention of PCOS to prevent the harmful effects of the disease on reproductive health. Researchers should also focus on the effects of PCOS on fertility in women with T2DM. Large-scale, multi-center, and high-quality clinical studies are required in the future.

In terms of the diagnostic criteria, it is recommended to consider the effects of different regions. More clinical studies are urgently needed to determine the impact of BMI, duration of T2DM, and treatment on the prevalence of PCOS to develop individualized screening and prevention strategies for a specific population.

## Data availability statement

The datasets presented in this study can be found in online repositories. The names of the repository/repositories and accession number(s) can be found in the article/**Supplementary Material**.

## Author contributions

CL, RY, and YL designed the study and drafted the manuscript. CL and RY systematically retrieved the literature and extracted data. CL reviewed the included studies and performed statistical analyses. HF, XC, WD, and YZ provided useful suggestions and substantial revisions based on the content of the article. All authors participated in the drafting of the manuscript and approved the final version.

## Funding

The study was supported by Sichuan Science and Technology Foundation (2021YFS0037) and Sichuan Provincial Administration of Traditional Chinese Medicine (2020LC0153).

## Conflict of interest

The authors declare that the research was conducted in the absence of any commercial or financial relationships that could be construed as a potential conflict of interest.

## Publisher’s note

All claims expressed in this article are solely those of the authors and do not necessarily represent those of their affiliated organizations, or those of the publisher, the editors and the reviewers. Any product that may be evaluated in this article, or claim that may be made by its manufacturer, is not guaranteed or endorsed by the publisher.
