# Supplementary material for: Prevalence of polycystic ovary syndrome in patients with type 2 diabetes: A systematic review and meta-analysis
Source: Front Endocrinol (Lausanne). 2022 Aug 31;13:980405. doi: 10.3389/fendo.2022.980405 (PMC9471325; doi:10.3389/fendo.2022.980405)
Supplement: Supplementary file 1 [file DataSheet_1.pdf]

Supplementary Material

1 SUPPLEMENTARY DATA

1.1 SUPPLEMENTARY MATERIAL 1: SEARCH STRATEGY

eTable 1. Search Strategy: PubMed

eTable 2. Search Strategy: Embase

eTable 3. Search Strategy: Cochrane Library

eTable 4. Search Strategy: Web of Science

1.2 SUPPLEMENTARY MATERIAL 2

eTable 5. Agency for healthcare research and quality (AHRQ) checklist (cross-sectional) for studies

1.3 SUPPLEMENTARY MATERIAL 3

eTable 6. Risk of Bias of Included Studies

1 SUPPLEMENTARY DATA

1.1 SUPPLEMENTARY MATERIAL 1: SEARCH STRATEGY

eTable 1. Search Strategy: PubMed

1	"Polycystic Ovary Syndrome"[MeSH Terms]
2	(((((((((((((Ovary Syndrome, Polycystic[Title/Abstract]) OR (Syndrome, Polycystic Ovary[Title/Abstract])) OR (Stein-Leventhal Syndrome[Title/Abstract])) OR (Stein Leventhal Syndrome[Title/Abstract])) OR (Syndrome, Stein-Leventhal[Title/Abstract])) OR (Sclerocystic Ovarian Degeneration[Title/Abstract])) OR (Ovarian Degeneration, Sclerocystic[Title/Abstract])) OR (Sclerocystic Ovary Syndrome[Title/Abstract])) OR (Polycystic Ovarian Syndrome[Title/Abstract])) OR (Ovarian Syndrome, Polycystic[Title/Abstract])) OR (Polycystic Ovary Syndrome 1[Title/Abstract])) OR (Sclerocystic Ovaries[Title/Abstract])) OR (Ovary, Sclerocystic[Title/Abstract])) OR (Sclerocystic Ovary[Title/Abstract])
3	#1 OR #2
4	"diabetes mellitus, type 2"[MeSH Terms]
5	(((((((((((((((((((((((((((((((Diabetes Mellitus, Noninsulin-Dependent[Title/Abstract]) OR (Diabetes Mellitus, Ketosis-Resistant[Title/Abstract])) OR (Diabetes Mellitus, Ketosis Resistant[Title/Abstract])) OR (Ketosis-Resistant Diabetes Mellitus[Title/Abstract])) OR (Diabetes Mellitus, Non Insulin Dependent[Title/Abstract])) OR (Diabetes Mellitus, Non-Insulin-Dependent[Title/Abstract])) OR (Non-Insulin-Dependent Diabetes Mellitus[Title/Abstract])) OR (Diabetes Mellitus, Stable[Title/Abstract])) OR (Stable Diabetes Mellitus[Title/Abstract])) OR (Diabetes Mellitus, Type II[Title/Abstract])) OR (NIDDM[Title/Abstract])) OR (Diabetes Mellitus, Noninsulin Dependent[Title/Abstract])) OR (Diabetes Mellitus, Maturity-Onset[Title/Abstract])) OR (Diabetes Mellitus, Maturity Onset[Title/Abstract])) OR (Maturity-Onset Diabetes Mellitus[Title/Abstract])) OR (Maturity Onset Diabetes Mellitus[Title/Abstract])) OR (MODY[Title/Abstract])) OR (Diabetes Mellitus, Slow-Onset[Title/Abstract])) OR (Diabetes Mellitus, Slow Onset[Title/Abstract])) OR (Slow-Onset Diabetes Mellitus[Title/Abstract])) OR (Type 2 Diabetes Mellitus[Title/Abstract])) OR (Noninsulin-Dependent Diabetes Mellitus[Title/Abstract])) OR (Noninsulin Dependent Diabetes Mellitus[Title/Abstract])) OR (Maturity-Onset Diabetes[Title/Abstract])) OR (Diabetes, Maturity-Onset[Title/Abstract])) OR (Maturity Onset Diabetes[Title/Abstract])) OR (Type 2 Diabetes[Title/Abstract])) OR (Diabetes, Type 2[Title/Abstract])) OR (Diabetes Mellitus, Adult-Onset[Title/Abstract])) OR (Adult-Onset Diabetes Mellitus[Title/Abstract])) OR (Diabetes Mellitus, Adult Onset[Title/Abstract]))
6	#4 OR #5
7	#3 AND #6

eTable 2. Search Strategy: Embase

1	'non insulin dependent diabetes mellitus'/exp
2	'diabetes mellitus, noninsulin-dependent':ab,ti
3	'diabetes mellitus, ketosis-resistant':ab,ti
4	'diabetes mellitus, ketosis resistant':ab,ti
5	'ketosis-resistant diabetes mellitus':ab,ti
6	'diabetes mellitus, non insulin dependent':ab,ti
7	'diabetes mellitus, non-insulin-dependent':ab,ti
8	'non-insulin-dependent diabetes mellitus':ab,ti
9	'diabetes mellitus, stable':ab,ti
10	'stable diabetes mellitus':ab,ti
11	'diabetes mellitus, type ii':ab,ti
12	'niddm':ab,ti
13	'diabetes mellitus, noninsulin dependent':ab,ti
14	'diabetes mellitus, maturity-onset':ab,ti
15	'diabetes mellitus, maturity onset':ab,ti
16	'maturity-onset diabetes mellitus':ab,ti
17	'maturity onset diabetes mellitus':ab,ti
18	'mody':ab,ti
19	'diabetes mellitus, slow-onset':ab,ti
20	'diabetes mellitus, slow onset':ab,ti
21	'slow-onset diabetes mellitus':ab,ti
22	type 2 diabetes mellitus':ab,ti
23	'noninsulin-dependent diabetes mellitus':ab,ti
24	.'noninsulin dependent diabetes mellitus':ab,ti
25	'maturity-onset diabetes':ab,ti
26	diabetes, maturity-onset':ab,ti
27	'maturity onset diabetes':ab,ti
28	type 2 diabetes':ab,ti
29	'diabetes, type 2':ab,ti
30	diabetes mellitus, adult-onset':ab,ti
31	'adult-onset diabetes mellitus':ab,ti
32	'diabetes mellitus, adult onset':ab,ti
33	#1 OR #2 OR #3 OR #4 OR #5 OR #6 OR #7 OR #8 OR #9 OR #10 OR #11 OR #12 OR #13 OR #14 OR #15 OR #16 OR #17 OR #18 OR #19 OR #20 OR #21 OR #22 OR #23 OR #24 OR #25 OR #26 OR #27 OR #28 OR #29 OR #30 OR #31 OR #32

34	ovary polycystic disease'/exp
35	'ovary syndrome, polycystic':ab,ti
36	'stein-leventhal syndrome':ab,ti
37	'stein leventhal syndrome':ab,ti
38	'syndrome, stein-leventhal':ab,ti
39	'sclerocystic ovarian degeneration':ab,ti
40	'ovarian degeneration, sclerocystic':ab,ti
41	'sclerocystic ovary syndrome':ab,ti
42	polycystic ovarian syndrome':ab,ti
43	'ovarian syndrome, polycystic':ab,ti
44	polycystic ovary syndrome 1':ab,ti
45	'sclerocystic ovaries':ab,ti
46	'ovary, sclerocystic':ab,ti
47	'sclerocystic ovary':ab,ti
48	#34 OR #35 OR #36 OR #37 OR #38 OR #39 OR #40 OR #41 OR #42 OR #43 OR #44 OR #45 OR #46 OR #47
49	#33 AND #48

eTable 3. Search Strategy: Cochrane Library

1	MeSH descriptor: [Diabetes Mellitus, Type 2] explode all trees
2	(Diabetes Mellitus, Noninsulin-Dependent):ti,ab,kw OR (Diabetes Mellitus, Ketosis-Resistant):ti,ab,kw OR (Diabetes Mellitus, Ketosis Resistant):ti,ab,kw OR (Ketosis-Resistant Diabetes Mellitus):ti,ab,kw OR (Diabetes Mellitus, Non Insulin Dependent):ti,ab,kw
3	(Diabetes Mellitus, Non-Insulin-Dependent):ti,ab,kw OR (Non-Insulin-Dependent Diabetes Mellitus):ti,ab,kw OR (Diabetes Mellitus, Stable):ti,ab,kw OR (Stable Diabetes Mellitus):ti,ab,kw OR (Diabetes Mellitus, Type II):ti,ab,kw
4	(NIDDM):ti,ab,kw OR (Diabetes Mellitus, Noninsulin Dependent):ti,ab,kw OR (Diabetes Mellitus, Maturity-Onset):ti,ab,kw OR (Diabetes Mellitus, Maturity Onset):ti,ab,kw OR (Maturity-Onset Diabetes Mellitus):ti,ab,kw
5	(Maturity Onset Diabetes Mellitus):ti,ab,kw OR (MODY):ti,ab,kw OR (Diabetes Mellitus, Slow-Onset):ti,ab,kw OR (Diabetes Mellitus, Slow Onset):ti,ab,kw OR (Slow-Onset Diabetes Mellitus):ti,ab,kw
6	(Type 2 Diabetes Mellitus):ti,ab,kw OR (Noninsulin-Dependent Diabetes Mellitus):ti,ab,kw OR (Noninsulin Dependent Diabetes Mellitus):ti,ab,kw OR (Maturity-Onset Diabetes):ti,ab,kw OR (Diabetes, Maturity-Onset):ti,ab,kw
7	(Maturity Onset Diabetes):ti,ab,kw OR (Type 2 Diabetes):ti,ab,kw OR (Diabetes, Type 2):ti,ab,kw OR (Diabetes Mellitus, Adult-Onset):ti,ab,kw OR (Adult-Onset Diabetes Mellitus):ti,ab,kw
8	(Diabetes Mellitus, Adult Onset):ti,ab,kw
9	#1 OR #2 OR #3 OR #4 OR #5 OR #6 OR #7 OR #8
10	MeSH descriptor: [Polycystic Ovary Syndrome] explode all trees
11	(Ovary Syndrome, Polycystic):ti,ab,kw OR (Syndrome, Polycystic Ovary):ti,ab,kw OR (Stein-Leventhal Syndrome):ti,ab,kw OR (Stein Leventhal Syndrome):ti,ab,kw OR (Syndrome, Stein-Leventhal):ti,ab,kw
12	(Sclerocystic Ovarian Degeneration):ti,ab,kw OR (Ovarian Degeneration, Sclerocystic):ti,ab,kw OR (Sclerocystic Ovary Syndrome):ti,ab,kw OR (Polycystic Ovarian Syndrome):ti,ab,kw OR (Ovarian Syndrome, Polycystic):ti,ab,kw
13	(Polycystic Ovary Syndrome 1):ti,ab,kw OR (Sclerocystic Ovaries):ti,ab,kw OR (Ovary, Sclerocystic):ti,ab,kw OR (Sclerocystic Ovary):ti,ab,kw
14	#10 OR #11 OR #12 OR #13
15	#9 AND #14

eTable 4. Search Strategy: Web of Science

1	((((((((((((((((((((((((((((((TS=(Diabetes Mellitus, Type 2)) OR TS=(Diabetes Mellitus, Noninsulin-Dependent)) OR TS=(Diabetes Mellitus, Ketosis-Resistant)) OR TS=(Diabetes Mellitus, Ketosis Resistant)) OR TS=(Ketosis-Resistant Diabetes Mellitus)) OR TS=(Diabetes Mellitus, Non Insulin Dependent)) OR TS=(Diabetes Mellitus, Non-Insulin-Dependent)) OR TS=(Non-Insulin-Dependent Diabetes Mellitus)) OR TS=(Diabetes Mellitus, Stable)) OR TS=(Stable Diabetes Mellitus)) OR TS=(Diabetes Mellitus, Type II)) OR TS=(NIDDM)) OR TS=(Diabetes Mellitus, Noninsulin Dependent)) OR TS=(Diabetes Mellitus, Maturity-Onset)) OR TS=(Diabetes Mellitus, Maturity Onset)) OR TS=(Maturity-Onset Diabetes Mellitus)) OR TS=(Maturity Onset Diabetes Mellitus)) OR TS=(MODY)) OR TS=(Diabetes Mellitus, Slow-Onset)) OR TS=(Diabetes Mellitus, Slow Onset)) OR TS=(Type 2 Diabetes Mellitus)) OR TS=(Noninsulin-Dependent Diabetes Mellitus)) OR TS=(Noninsulin Dependent Diabetes Mellitus)) OR TS=(Maturity-Onset Diabetes)) OR TS=(Diabetes, Maturity-Onset)) OR TS=(Maturity Onset Diabetes)) OR TS=(Type 2 Diabetes)) OR TS=(Diabetes, Type 2)) OR TS=(Diabetes Mellitus, Adult-Onset)) OR TS=(Adult-Onset Diabetes Mellitus)) OR TS=(Diabetes Mellitus, Adult Onset)
2	((((((((((((((TS=(Polycystic Ovary Syndrome)) OR TS=(Ovary Syndrome, Polycystic)) OR TS=(Syndrome, Polycystic Ovary)) OR TS=(Stein-Leventhal Syndrome)) OR TS=(Stein Leventhal Syndrome)) OR TS=(Syndrome, Stein-Leventhal)) OR TS=(Sclerocystic Ovarian Degeneration)) OR TS=(Ovarian Degeneration, Sclerocystic)) OR TS=(Sclerocystic Ovary Syndrome)) OR TS=(Polycystic Ovarian Syndrome)) OR TS=(Ovarian Syndrome, Polycystic)) OR TS=(Polycystic Ovary Syndrome 1)) OR TS=(Sclerocystic Ovaries)) OR TS=(Ovary, Sclerocystic)) OR TS=(Sclerocystic Ovary)
3	#1 AND #2

1.2 SUPPLEMENTARY MATERIAL 2

eTable 5. Agency for healthcare research and quality (AHRQ) checklist (cross-sectional) for studies

Study	Items scored											Total score	Quality
	1	2	3	4	5	6	7	8	9	10	11		
Amed2011	1	0	0	1	0	1	0	1	0	1	0	5	Moderate
Amed2012	1	0	0	1	1	1	0	0	0	1	0	5	Moderate
Amini2008	1	1	0	1	1	0	1	1	0	0	0	6	Moderate
Amutha2012	1	0	0	1	0	0	0	0	0	0	0	2	Low
Balasanthiran2011*	1	0	0	0	0	0	0	0	0	0	0	1	Low
Kelestimur2006	1	1	0	0	1	0	1	1	1	1	0	7	Moderate
Mirzaei2008	1	1	0	1	1	0	0	1	0	0	0	5	Moderate
Sim2016	1	1	0	1	1	1	1	1	0	1	0	8	High
Zargar2005	1	1	0	1	1	1	0	0	0	0	0	5	Moderate
Peppard2001	1	1	0	1	1	0	1	1	1	1	0	8	High
Zdravkovic2004	1	0	0	1	1	0	0	0	0	0	0	3	Low
Ramachandran2003	1	1	0	1	0	0	0	0	0	0	0	3	Low
Shield2009	1	0	0	1	1	1	1	0	0	1	1	7	Moderate
Wilmot2010*	1	0	0	0	0	0	0	0	0	0	0	1	Low

Footnote:*=Conference paper; 0: no, 1: yes, overall risk of bias: low (score >8), moderate (score 6-8), or high (score ≤5). Items scored: 1) Define the source of information (survey, record review); 2) List inclusion and exclusion criteria for exposed and unexposed subjects (cases and controls) or refer to previous publications; 3) Indicate time period used for identifying patients; 4) Indicate whether or not subjects were consecutive if not population-based; 5) Indicate if evaluators of subjective components of study were masked to other aspects of the status of the participants; 6) Describe any assessments undertaken for quality assurance purposes (e.g., test/retest of primary outcome measurements); 7)Explain any patient exclusions from analysis; 8) Describe how confounding was assessed and/or controlled; 9) If applicable, explain how missing data were handled in the analysis; 10) Summarize patient response rates and completeness of data collection; 11) Clarify what follow-up, if any, was expected and the percentage of patients for which incomplete data or follow-up was obtained.

1.3 SUPPLEMENTARY MATERIAL 3

eTable 6. Risk of Bias of Included Studies

Study	External Validity Items					Internal Validity Items					Overall Score	Overall Risk of Bias
	1	2	3	4	5	6	7	8	9	10		
Amed2011	1	0	0	1	1	0	0	1	1	1	6	Moderate
Amed2012	1	0	0	1	1	0	0	1	1	1	6	Moderate
Amini2008	0	1	1	1	1	0	0	1	1	1	7	Moderate
Amutha2012	0	0	1	1	1	0	0	1	1	1	6	Moderate
Balasanthiran2011*	0	0	0	0	1	0	0	1	0	0	2	High
Kelestimur2006	0	0	0	0	1	0	0	1	1	1	4	High
Mirzaei2008	0	0	0	0	1	1	1	1	1	1	6	Moderate
Sim2016	0	0	0	0	1	1	1	1	1	1	6	Moderate
Zargar2005	0	0	0	0	1	1	0	1	1	1	5	High
Peppard2001	0	1	1	1	1	1	0	1	1	1	8	Moderate
Zdravkovic2004	0	1	1	1	1	1	1	1	1	1	9	Low
Ramachandran2003	0	0	0	0	1	0	0	0	1	1	3	High
Shield2009	1	1	1	1	1	0	1	1	1	1	9	Low
Wilmot2010*	0	0	0	0	1	0	0	0	1	1	3	High

Footnote: * = Conference paper; 0: no, 1: yes, overall risk of bias: low (score > 8), moderate (score 6 - 8), or high (score ≤ 5). Items scored: 1) Was the study's target population a close representation of the national population in relation to relevant variables, e.g., age, sex?; 2) Was the sampling frame a true or close representation of the target population?; 3) Was some form of random selection used to select the sample, OR, was a census undertaken?; 4) Was the likelihood of non-response bias minimal?; 5) Were data collected directly from the subjects (as opposed to a proxy)?; 6) Was an acceptable case definition used in the study?; 7) Had the study instrument that measured the parameter of interest (e.g., prevalence of comorbidity) been tested for reliability and validity (if necessary)?; 8) Was the same mode of data collection used for all subjects?; 9) Was the length of the shortest prevalence period for the parameter of interest appropriate?; 10) Were the numerator(s) and denominator(s) for the parameter of interest appropriate?
